# Domestic Bull Crossing with the Buffalo Cow

**Published:** 1912-12

**Authors:** Silas Hubbard

**Affiliations:** East Aurora, N. Y.


### Domestic Bull Crossing with the Buffalo Cow

I was the first to show by certain new theories how males and females are produced, as can be seen on page 645 of Vol. X of the BUFFALO MEDICAL JOURNAL, 1855, and now I would add to this that I have observed that crossing the domestic bull with the American bison almost invaribly produces females. I will say that the domestic bull is so fearful of the Buffalo cow that he neglects the Buffalo cow till the ovum becomes very mature and thus the products are more likely to be females, for the very mature impregnated ova are more likely to develop into females, and the more immature ova develop into males.

SILAS HUBBARD, M. D.,
East Aurora, N. Y.

NOTE.—It is not often that a Journal has the pleasure of supplementing an article by another communication from the same author 57 years later. Dr. Hubbard's original article is an elaborate study of cross breeding, well worth our attention and we shall be pleased to allow anyone interested to refer to it. Dr. Hubbard graduated in medicine, 70 years ago, at the Castleton Medical College, Vermont. This institution was chartered in 1818 and was discontinued in 1861. It graduated 350 students.

